# Ultrastructural cellular signatures: does cellular form follow function?

**DOI:** 10.1093/nsr/nwz057

**Published:** 2019-05-16

**Authors:** Manfred Auer

**Affiliations:** Molecular Biophysics and Integrated Bioimaging Division, Lawrence Berkeley National Laboratory, USAE-mail: mauer@lbl.gov

In his watershed 1896 essay *The Tall Office Building Artistically Considered*, protomodern architect Louis Sullivan famously declared that ‘form follows function’. Could this concept also hold true in biology? Have cells, through evolutionary optimization, adopted a 3D architecture (‘form’) that reflects their function? Or, to keep with the architectural metaphor, how does ‘form’ relate to the cell's ‘intended function or purpose’? Is there an ultrastructural signature that reflects the ‘constraints and requirements’ of a cell's function in its specific spatial and temporal context?

To be fair, cells of similar function will very likely never display the exact same ultrastructural 3D organization. Like in architecture and design there is not one single solution, but instead a variety of 3D organizational solutions that satisfy the constraints of function. Just as the many houses in any given city are different from one another, there are also many shared similarities such as doors, windows, roofs and walls. And just like office buildings in their function and layout differ from single-home residences, so will cells that face different ‘functional constraints and requirements’, even though they use the same components and display an overall similar inventory.

For example, epithelial cells—independent of the organ they reside in—are highly similar to one another, e.g. in terms of cell polarity, cell–cell adhesion and the 3D organization of cytoskeletal filaments. Epithelial cells (Fig. 1A) are distinct from other cells that constitute nervous, muscle or connective tissue, or from individual cells that migrate through the extracellular matrix (Fig. 1B). Moreover, each cell type will undergo changes throughout their life cycle. Some mammary gland epithelial cells reside already in a tissue-like context before they undergo branching morphogenesis (Fig. 1C), e.g. during organ development [1] before they differentiate and undergo senescence. Yet, other mammary gland epithelial cells will transform from a healthy to a premalignant (Fig. 1D) and malignant (Fig. 1E) diseased stage. For each of all these different stages, the cells will display unique patterns of 3D organization of organelles, like the nucleus, mitochondria and endoplasmic reticulum (Fig. 1F), supramolecular complexes such as microvilli-like membrane protrusions (Fig. 1G), cell–cell adhesion sites/desmosomes (Fig. 1H) and the cytoskeleton (Fig. 1I) (see also [2]).

**Figure 1:**
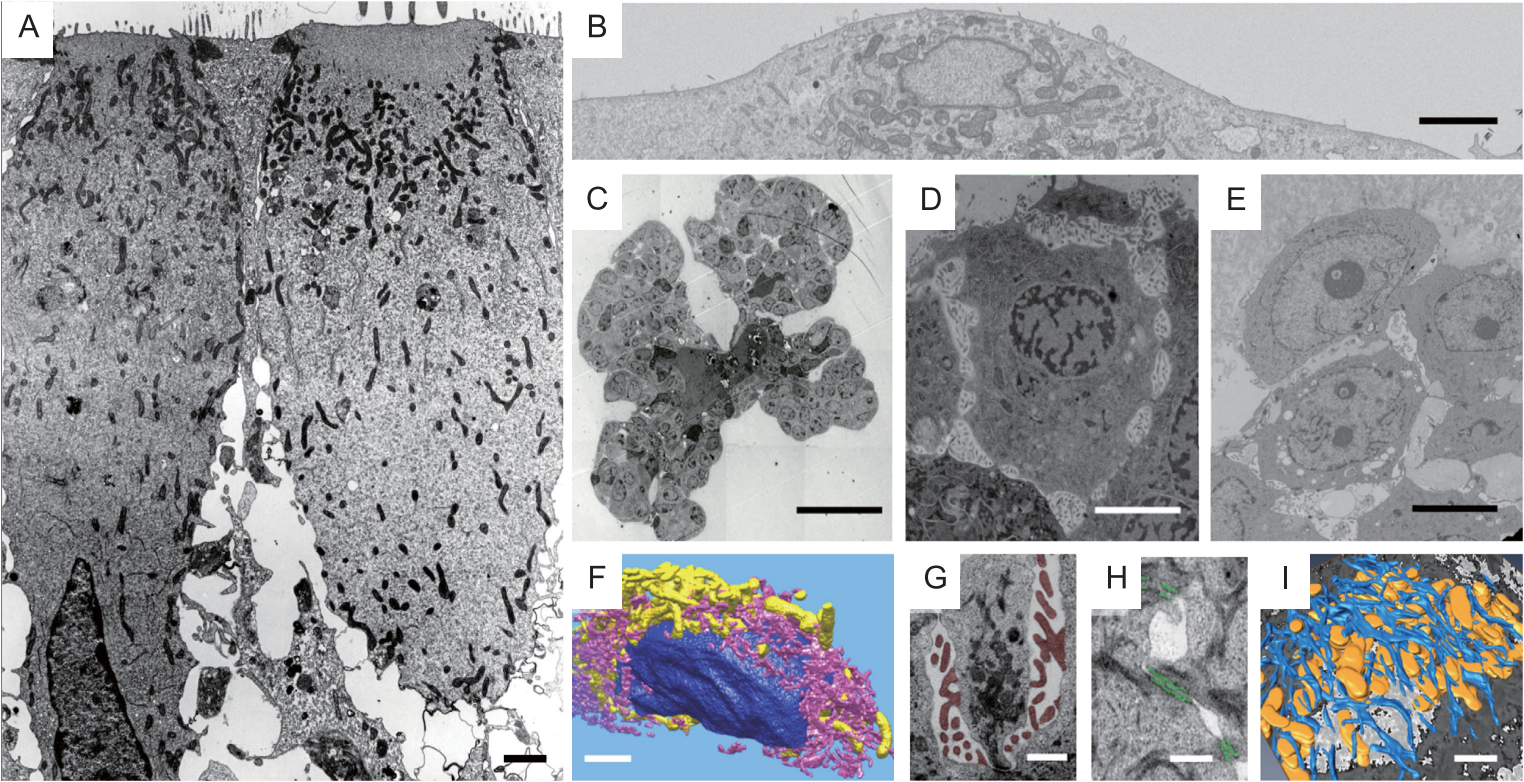
Ultrastructure of cells in different cell and tissue contexts. (A) 2D TEM projection view inner of a sensory epithelium reveals apical–basolateral polarity, with its characteristic distribution of apical microvilli and basolateral cell–cell adhesion complexes, which are separated by tight junction complexes that seal off the luminal space from the basolateral space. (B) Block-surface SEM image of an individual fibrosarcoma cell attached to a substrate surface. (C) Murine mammary gland cells forming organoids, undergoing FGF2-induced branching morphogenesis. (D) Human mammary gland cells grown in a 3D matrix, forming premalignant, growth-arrested acini. Note the sparse cell–cell adhesion and prominent presence of microvilli-like apical cell protrusions, which are both reminiscent of the cell aggregates undergoing branching morphogenesis. (E) Mammary gland cells grown in a 3D matrix forming malignant cell aggregates, which are no longer growth-arrested and do possess metastatic potential. (F) 3D rendering of segmented nucleus (color: navy blue), endoplasmic reticulum (color: salmon) and mitochondria (color: yellow) of 3D data set shown in (B). (G) Segmented microvilli-like protrusions residing between adjacent cells, frequently flanked on both sides by microvilli, typically seen in both (C) and (D), and to some extent in (E), but not in mature epithelial tissues. (H) Desmosomes (color: lime green) connecting adjacent cell–cell adhesion complexes. (I) 3D rendering of the cytoskeleton (color: blue) and mitochondria (color: golden) of data sets similar to the one shown in (D). Scale bars: (A, B) 2 μm, (C) 50 μm, (D, E) 5 μm and (F–I) 1 μm.

One may argue that the combination of their respective abundance, size, shape, 3D distribution and other characteristics constitutes an ‘ultrastructural fingerprint’ (see Fig. 2) or a ‘cellular signature’ that uniquely characterizes each of these cells in their respective spatiotemporal context. If one accepts this premise, how does one go about determining such ‘cellular signatures’?

For centuries, observation of structure has been key for an understanding of life, first at the levels of anatomy, then histology/neuroanatomy, and more recently cell biology and biochemistry. The two disciplines that study of the organization of cells and the 3D structures of proteins are called structural cell biology and structural molecular biology, respectively. Structural biology in its classical sense aims to determine the precise shapes of proteins and other macromolecules, ideally at atomic resolution. In a well-received commentary called *Whither Structural Biology*, Steven Harrison [3] described the state of the art in 3D-structure determination and laid out a path to expand structural biology to the highly regulated complexity of cells, e.g. through the further development of cryotomography and computational modeling. However, in this vision, the goal of structural cell biology remains the visualization of cellular sceneries, albeit at a somewhat lower resolution, and to identify and then dock protein and/or protein complex structures into such cryotomography density maps.

In the last four decades, structural molecular biology [4] has strongly outperformed structural cell biology [5–7] through implicit or explicit averaging by X-ray crystallography, and more recently by cryoelectron microscopy, culminating in the 2017 Nobel Prize in Chemistry. While tens of thousands of atomic structures are known, only a few attempts have been made to map their precise location inside a cell's ultrastructural density map [8,9], in part due to the fact that only very large complexes can be unambiguously identified without the need for a label. Correlative light and electron microscopy approaches aim to superimpose fluorescence signals onto an electron density map [10,11]; however, a variety of technical challenges have kept the success rate to date somewhat low.

To be clear, what is proposed here is not a classical structural biology approach. Instead, the goal here is to identify which of several categories a certain ultrastructural feature belongs to, e.g. to the category of cell–cell adhesion complexes. Categorization is followed by the determination of its abundance and 3D localization, by measurements of its dimensions (i.e. the cell–cell contact size) as well as the number and 3D organization of attached cytoskeletal filaments. The relative strength of cell adhesion for respective cells can be inferred from these parameters without the knowledge of any atomic-resolution model for the individual proteins involved. Likewise, for the cytoskeleton, one may want to determine its abundance and distribution, the degree of cross-connectivity and the regularity of its pattern, as well as the abundance, distribution and type of all of its interaction partners (such as the nucleus or adhesion complexes). Such cytoskeletal signatures may eventually serve as a starting point for the computational simulation of mechanical properties under different 3D architectural scenarios [12].

Given that every cell is unique, one key challenge is to discriminate truly different signatures from the heterogeneity inevitably inherent in biological systems. A promising route may be a rigorous statistical analysis, which in turn requires large-scale 3D data sets, ideally of entire cells and small tissues/organoids at a resolution that allows the simultaneous identification of the major supramolecular assemblies and organelles.

Focused Ion Beam Scanning Electron Microscopy is an ideal technique for imaging such large cellular volumes [13].

Apart from instrument access and data acquisition time, there are no limits on the size of volume it can cover at a resolution of ∼7–10 nm. Such intermediate resolution will be too coarse to identify individual proteins by shape alone (which is not the goal of cellular imaging anyway), but allows the discrimination of supramolecular complexes such as the cytoskeleton and cell–cell adhesion complexes, as well as organelles. With careful (e.g. cryogenic) sample preparation methods that avoid artifacts such as aggregation and extraction, cells retain the 3D organization of their supramolecular complexes and organelles, even when samples are subjected to heavy metal staining, dehydration and resin-embedding [14].

The challenge of turning voxel information into knowledge arises in part from the size of the data sets, which can reach tens of terabytes, and in part from the high feature density combined with the high complexity of cellular constituent supramolecular complexes and organelles, which are in close contact with one another. Supervised machine learning, also known as deep learning, requires a large amount of so-called ‘ground truth’ that typically needs to be established manually (and thus painstakingly) by a human expert. Deep learning currently lacks the accuracy that is needed to fully automate the process of segmentation. Deep learning may also be employed to assess the similarities between different cells and discriminate cellular signatures from sample heterogeneity.

**Figure 2:**
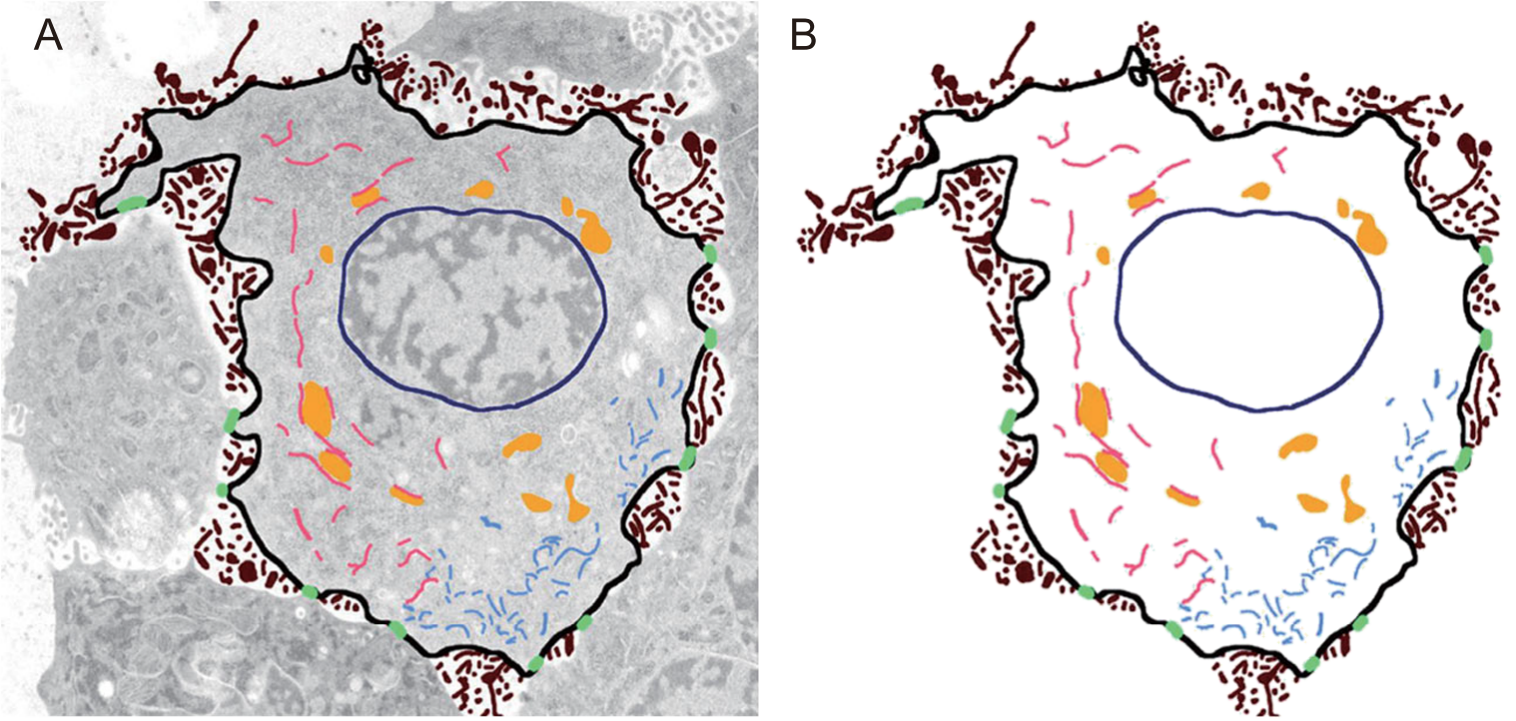
Ultrastructural signature of cellular state. (A) Superposition of original image data with manually segmented image data (B). Note that the plasma membrane is shown in black, microvilli-like protrusions are shown in brown, nuclear membrane is shown in navy blue, endoplasmic reticulum is shown in salmon, mitochondria are shown in a golden color, the cytoskeleton is shown in light blue and desmosome-like cell–cell adhesion complexes are shown in lime green.

Furthermore, feature extraction and classification is only the starting point for turning voxels into knowledge. To reach that ultimate goal, resolution-faithful simplified volumetric models of supramolecular complexes and organelles need to be built automatically, and subjected to rigorous measurements of volumetric properties. Such a comprehensive quantitative statistical analysis will allow the creation of prototypic models that acknowledge and represent the ultrastructural heterogeneity inherent in biological data sets. Also missing is a semantic mathematical description of cellular 3D architecture, capable of providing the respective relationships of constituents, and their hierarchy. Last but not least, prototypic models ideally will reside in map-centered ‘biospatial’ databases, not unlike ‘Google Maps’, which then could be queried for patterns. Such a ‘Google-Cell Biology’ database would become the platform to integrate—at the model level—additional imaging, metabolic or proteomics data, as well as encyclopedic knowledge.

In summary, what I am proposing here builds on, yet goes beyond, traditional structural molecular and cellular biology. Instead of determining the 3D structure of individual macromolecules or cellular sceneries, the objective is to quantify the geometric properties of simplified volumetric models. Instead of determining a single, defined structure, we seek for each cellular state to identify the range of such geo/volumetric model properties with sufficient commonalities to constitute an ultrastructural cellular signature. Such cellular signatures will reflect the ‘form’ and the ultrastructural heterogeneity that is inherent in each state, and will allows us to discriminate heterogeneity from truly altered states and thus yield mechanistic insight into the cell's ‘intended function or purpose’.

## References

[1] Ewald AJ , Huebner RJ and Palsdottir H et al. J Cell Sci 2012; 125: 2638–54. 22344263 10.1242/jcs.096875 PMC3403234

[2] Jorgens DM , Inman JL and Wojcik M et al. J Cell Sci 2017; 130: 177–89. 27505896 10.1242/jcs.190967 PMC5394780

[3] Harrison SC . Nat Struct Mol Biol 2004; 11: 12–5. 14718917 10.1038/nsmb0104-12

[4] Cheng Y . Science 2018; 361: 876–80. 30166484 10.1126/science.aat4346 PMC6460916

[5] Koning RI , Koster AJ and Sharp TH. Ann Anat 2018; 217: 82–96. 29526767 10.1016/j.aanat.2018.02.004

[6] Pfeffer S and Mahamid J. Curr Opin Struct Biol 2018; 52: 111–8. 30339965 10.1016/j.sbi.2018.08.009

[7] Weber MS , Wojtynek M and Medalia O. Cells 2019; 8: E57 30654455 10.3390/cells8010057 PMC6356268

[8] Asano S , Engel BD and Baumeister W. J Mol Biol 2016; 428: 332–43. 26456135 10.1016/j.jmb.2015.09.030

[9] Robinson CV , Sali A and Baumeister W. Nature 2007; 450: 973–82. 18075576 10.1038/nature06523

[10] Schorb M and Briggs JA. Ultramicroscopy 2014; 143: 24–32. 24275379 10.1016/j.ultramic.2013.10.015 PMC5472196

[11] Zhang P . Curr Opin Struct Biol 2013; 23: 763–70. 23962486 10.1016/j.sbi.2013.07.017 PMC3812453

[12] Fletcher DA and Mullins RD. Nature 2010; 463: 485–92. 20110992 10.1038/nature08908 PMC2851742

[13] Titze B and Genoud C. Biol Cell 2016; 108: 307–23. 27432264 10.1111/boc.201600024

[14] McDonald KL and Auer M. Biotechniques 2006; 41: 137, 139, 141, 143. 10.2144/000112226 16925014

